# Pediatric Health-Related Quality of Life: A Structural Equation Modeling Approach

**DOI:** 10.1371/journal.pone.0113166

**Published:** 2014-11-21

**Authors:** Ester Villalonga-Olives, Ichiro Kawachi, Josué Almansa, Claudia Witte, Benjamin Lange, Christiane Kiese-Himmel, Nicole von Steinbüchel

**Affiliations:** 1 Institute of Medical Psychology and Medical Sociology, Georg-August-University Göttingen, Göttingen, Germany; 2 Department of Social and Behavioral Sciences, Harvard School of Public Health, Boston, Massachusetts, United States of America; 3 Department of Health Sciences, Community and Occupational Medicine, University of Groningen, University Medical Center Groningen, Groningen, The Netherlands; National Institute of Genomic Medicine, Mexico

## Abstract

**Objectives:**

One of the most referenced theoretical frameworks to measure Health Related Quality of Life (HRQoL) is the Wilson and Cleary framework. With some adaptions this framework has been validated in the adult population, but has not been tested in pediatric populations. Our goal was to empirically investigate it in children.

**Methods:**

The contributory factors to Health Related Quality of Life that we included were symptom status (presence of chronic disease or hospitalizations), functional status (developmental status), developmental aspects of the individual (social-emotional) behavior, and characteristics of the social environment (socioeconomic status and area of education). Structural equation modeling was used to assess the measurement structure of the model in 214 German children (3–5 years old) participating in a follow-up study that investigates pediatric health outcomes.

**Results:**

Model fit was χ2 = 5.5; df = 6; p = 0.48; SRMR  = 0.01. The variance explained of Health Related Quality of Life was 15%. Health Related Quality of Life was affected by the area education (i.e. where kindergartens were located) and development status. Developmental status was affected by the area of education, socioeconomic status and individual behavior. Symptoms did not affect the model.

**Conclusions:**

The goodness of fit and the overall variance explained were good. However, the results between children' and adults' tests differed and denote a conceptual gap between adult and children measures. Indeed, there is a lot of variety in pediatric Health Related Quality of Life measures, which represents a lack of a common definition of pediatric Health Related Quality of Life. We recommend that researchers invest time in the development of pediatric Health Related Quality of Life theory and theory based evaluations.

## Introduction

To evaluate outcomes in terms of prevention, treatment and rehabilitation in children, it is important to test Health Related Quality of Life (HRQoL) [1]. Measures of HRQoL provide a broad view of child health, encompassing aspects of perceived health, health behavior, and well-being. Therefore, HRQoL has the potential to describe the health of children in the general and specific population more comprehensively than conventional health measures and provide better identification of specific groups with high rates of unrecognized conditions, social and emotional problems, and poor well-being and functioning [2]. Several variables have been identified as associated with HRQoL, including functional status, symptom status, biological status, and health perception [3].

The World Health Organization has proposed that the theoretical frameworks for conceptualizing the health of children and adults should be harmonized. One example is the International Classification of Functioning, Disability and Health (ICF) [4], in which the bio-psycho-social models for adults and children/youths do not vary. The WHO argues that what differs are the indicators for each component of the classifications. Differences between these populations have been summarized based on stage of human development, dependency, differential epidemiology, and demographics [5]. This means that, for example, the component body function of the ICF checklist for adults measures sexual functioning, while the ICF-CY (children/youths) measures genital functions. Another example would be in the activity limitations and performance component. There, language acquisition is an indicator in the checklist for children, whereas in the checklist version for adults, solving problem ability is emphasized.

One of the most referenced theoretical frameworks to measure HRQoL in the literature is the Wilson and Cleary model [3], [6]–[8], which presents a conceptual model, a taxonomy of patient outcomes that categorizes patient outcomes according to the underlying health concepts they represent; it proposes specific causal relationships between the different health concepts [9] and focuses on relationships among different domains of health; its principal goal is to specify a series of critical concepts along a causal pathway. It proposes a linear sequence of causal relationships that proceeds from biological/physiological perturbance → symptoms → function → perceptions → overall Health Related Quality of Life. The theoretical framework's implication is that researchers need to measure these various outcomes and develop statistical models that explicitly estimate the size of the effects specified within the model. Sousa et al. [7] tested the model in an adult population with HIV-associated illness, to validate it as suggested by the original authors, using Structural Equation Modeling. However, it has never been tested in the pediatric population.

Our aim was to investigate the applicability of the framework of Wilson and Cleary in children by empirically testing the dominant causal associations they propose, using pediatric data available in our study.

## Materials and Methods

### Participants

The participants of the study comprised the baseline data of a longitudinal study whose main aim was to investigate developmental outcomes and well-being in children from 3 to 5 years old with predominantly migrant backgrounds (second generation migrants).The study was conducted in five kindergartens in Frankfurt/Main and Darmstadt, Germany, located in different neighborhoods. Participants were enrolled at the kindergartens. The parents of 96% of the children enrolled in these kindergartens consented to their children taking part of the study (N = 220 children).

The project was approved by the local ethics committee of the University Medical Center Göttingen. Written informed consent was obtained from the participating families (directly from the parents), together with the approval of the kindergarten councils. Families received detailed information regarding the background and implementation of the study and were offered the opportunity to withdraw their children from the study at any time. No incentives were given.

### Investigation Tools and Questionnaires

#### Socioeconomic variables

We collected information about occupation and level of education of the main sustainer of the family to characterize the family's socioeconomic status. Here the international ISCO categorization was followed [10]. To perform the analysis of these data, we created a final categorical variable: out of work (0), unskilled workers (1), skilled workers (2), professionals (3) and professionals with advanced qualifications (4).

#### Kiddy-KINDL *(KK; Ravens-Sieberer et al., 1998)*

The “Kiddy-KINDL (KK)” is an instrument designed to measure general HRQoL in children aged between 4 and 7 [1], [11]. The recall period of the questionnaire is the past week. The short version of this questionnaire comprises 12 items belonging to 6 dimensions: physical well-being, psychological well-being, self-esteem, family, friends, and everyday functioning at the kindergarten. Response categories are arranged on a 3 point Likert scale (never, sometimes, very often). The final scores are T-scores that range from 20–80, with higher scores indicating better HRQoL. We used KK in interviews to collect self-reports from children who were 3–5 years old. We previously tested the psychometric properties of the instrument in the present sample and found their overall validity and overall reliability scores to be acceptable to very good. The work is under review elsewhere.

#### Symptom status

As an indicator of symptom status, we asked whether the child had recently been in a hospital or had a chronic disease (Table 1). Response categories were yes/no.

**Table 1:** Indicators used in the study to test the Wilson and Cleary theoretical framework in pediatric data: concepts, measured variables and details of the instruments used.

Concepts	Measured variables	Instrument	Recall period	Respondent	Content example
**HRQoL**	HRQoL	Kiddy-KINDL	Past week	Children	Had fun at the kindergarten
**Environmental factors**	Socioeconomic status	Specific questions	At present	Parents	Level of education, and current job
**Symptom status**	Symptom status	Specific question	Past week	Children	Recently been in a hospital or have a long disease
**Functional status**	Development status	WET	At present	Parents and Children	Put on the shoes, assists in housework
**Characteristics of the individual**	Individual Behavior	VBV 3–6 scale	Past four weeks	Kindergarten Teacher	Shows feelings spontaneously

#### Wiener Entwicklungstest *(WET - Vienna Development Test; Kastner-Koller and Deimann, 2002)*

The WET is a widely used instrument which measures the developmental status of children aged 3 to 6 years [12]. The WET consists of 13 subtests and a parent questionnaire, covering 6 functional areas of development: visual, motor, learning/memory, cognitive stage, language, and socio-emotional development, and a final score that measures overall development. The variables and the final score range from 0 to 9, with higher scores indicating better development. We used C scores in the analysis. The instrument has good face validity and construct validity [12].

#### Verhaltensbeurteilungsbogen für Vorschulkinder *(Behavioral Assessment Rating Scale for Preschool Children. VBV 3–6; M.Döpfner, W Berner, T. Fleischmann et al., 1993)*

The VBV is an observation- and rating scale for behavioral problems that has 93 items organized in 4 scales: social-emotional competence, oppositional-aggressive behavior, attention deficit/hyperactivity versus playing time and emotional disorders. Responses are arranged on a 5 point rating-scale (never; once a week; several times a week; every day and several times a day). A sum score for every scale and an overall sum score can be calculated and these can be transferred into stanine norm scores (ranging from 1 to 9) with higher scores indicating more appropriate behavior. In this study, only information about the social-emotional behavior was collected. Kindergarten teachers rated the scales based on observed behavior in the last 4 weeks. The psychometrical properties are acceptable [13].

In this study we have included area-level education in the model. This variable is considered an environmental factor because every kindergarten is located in a different neighborhood. We selected this variable to account for the potential contextual effect of neighborhoods [14]. We used the WET developmental score and the social-emotional behavior score of the VBV 3–6 scale. More information is provided at Table 1.

### Procedure

Data were collected at the kindergartens during day care. Children were interviewed and tested by psychologists and educational scientists. The same interviewers assessed the socioeconomic status of the family in an interview with the parents at the kindergartens. Information was gathered following time sequence of variables, and the information of the Kiddy-KINDL was reported between 10 and 15 days after the collection of the other variables.

### Adaptation of the Wilson and Cleary framework

**Figure 1:**
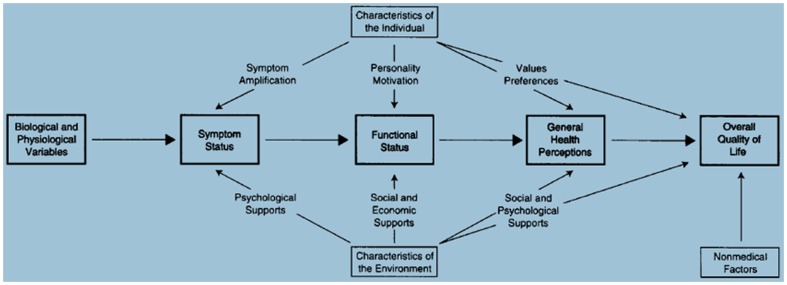
Wilson and Cleary theoretical framework.

**Figure 2:**
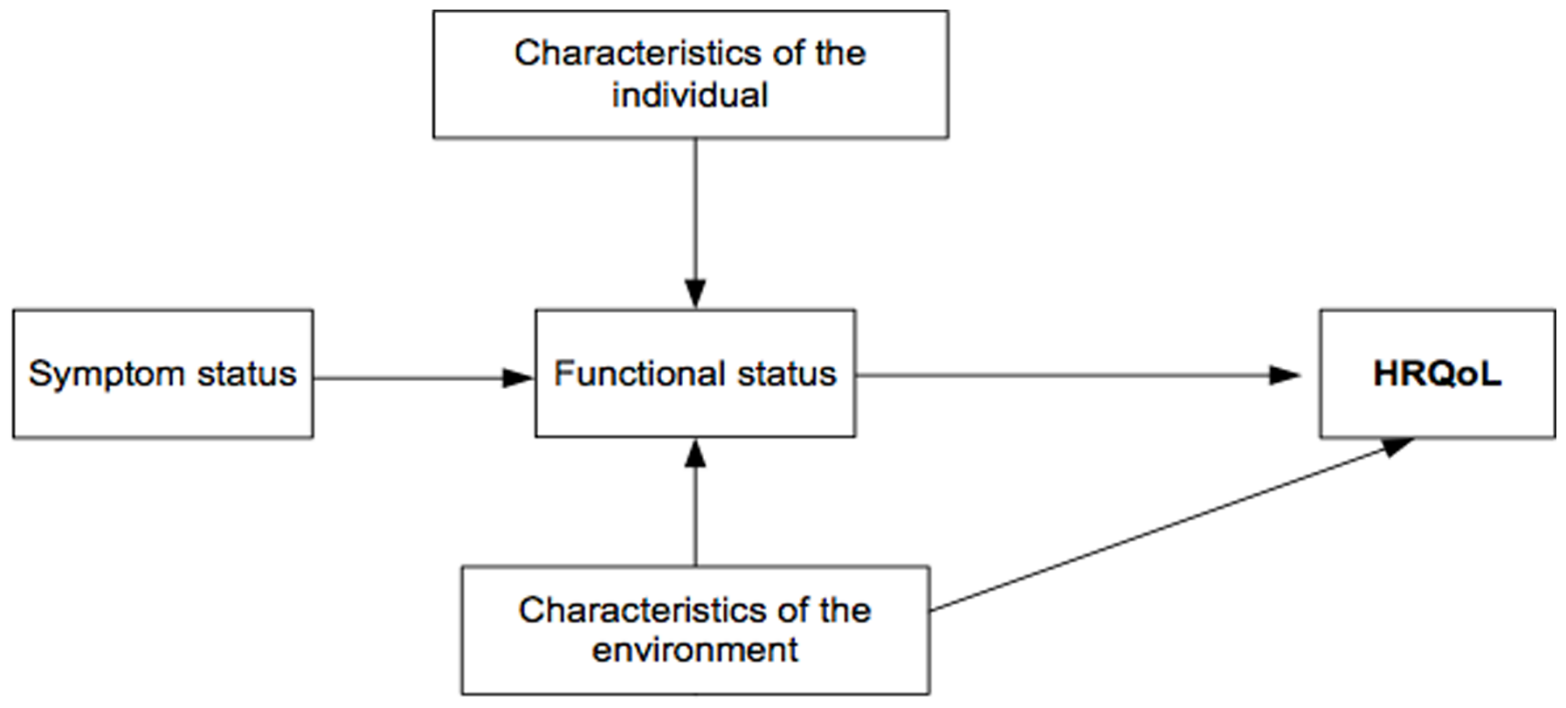
Structural model to test contributions to HRQoL in children. Adapted from the Wilson and Cleary theoretical framework.

Wilson and Cleary postulated that six categories of variables were directly or indirectly related to overall Quality of Life: health perception, symptom status, functional status, biological/physiologic status, characteristics of individual behavior, and environmental characteristics (Figure 1). We sought to test a reduced version of the model with the data available in our study: symptom status, functional status, individual and environmental characteristics (Figure 2). The variables ‘health perceptions’ and ‘biological/physiological factors’ were not obtainable from our data. Symptom status was collected using the information concerning hospital visits or having a chronic disease. In the case of functional status, we used the overall developmental score of the WET that was the sum of the six subscales: visual, motor, learning/memory, cognitive stage, language, and socio-emotional development. As a characteristic of the individual behavior we used the social-emotional behavior score of the VBV 3–6 scale. We also included the socioeconomic status of the family's main sustainer as well as the area of education as a measure of the environment. We tested the model with general HRQoL, since most of instruments that evaluate HRQoL measure general HRQoL and we tested a predominantly healthy group. The same relationships (arrows) that Wilson and Cleary model suggested (Figure 2) were hypothesized.

### Statistical Analyses

Structural equation modeling was used to assess the measurement structure of the model [15], [16]. Model fit was evaluated using χ2, and Standardized Root Mean Square Residual (SRMR) [17]. The values to accept the model should be non-significant in the χ2, and be lower than 0.07 in the SRMR. We divided the variable that measures HRQoL (Kiddy-KINDL) by 10 to obtain similar range of values across all variables, since it had a wide range scale (0–100), whereas the other instruments had ranges of 0–10 and 0–9. The socioeconomic variable and the area of education were introduced in the model with dummy coding. The reference category for the socioeconomic variable was unemployment. Modification indices were explored. For 214 children sufficient information was collected to be included in the analyses. Missing data were imputed by substituting for the missing value the scale mean rounded to an integer. Means for all instruments used in the analysis were calculated if up to 33% of responses were missing. The analyses were performed using maximum likelihood in M-Plus.

## Results

50.5% of the participants were boys. They reported a score of 66.70 (SD 17.33) in HRQoL, and girls 72.39 (SD 15.94). Regarding socioeconomic status,15% of the main sustainers of the children families were skilled manual workers, and 13.2% of them were unemployed. The 6.5% of the sample declared to have a chronic disease or having been hospitalized the last week. Developmental status overall score for boys was 4.27 (SD 1.00), while for girls was 4.71 (SD 1.26). In the case of individual behavior, the score for boys was 4.14 (SD 2.08) and 5.03 (SD 2.41) for girls. 214 children presented sufficient information to be included in the present analysis (Table 2).

**Table 2:** Descriptive statistics of the study sample.

	Overall N = 214	Boys N = 108	Girls N = 106
	Mean (SD) Percentage	Mean (SD) Percentage	Mean (SD) Percentage
**Age**	4.28 (1.47)	4.25 (1.57)	4.31 (1.37)
**HRQoL**	69.54 (16.63)	66.70 (17.33)	72.39 (15.94)
**Area of education(kindergarten 1 = St Gallus)**	29.0%	26.9%	31.1%
**Area of education (kindergarten 2 = St Pius)**	19.5%	23.1%	16.0%
**Area of education(kindergarten 3 = St Fidelis)**	19.6%	19.4%	19.8%
**Area of education(kindergarten 4 = St Martin)**	14.7%	13.5%	16.0%
**Area of education(kindergarten 5 = St Michael)**	16.8%	16.7%	17.0%
**Professionals with advanced qualifications**	17.2%	15.6%	18.8%
**Professionals**	11.1%	10.4%	11.8%
**Skilled workers**	43.5%	45.8%	41.2%
**Unskilled workers**	15.0%	13.5%	16.5%
**Unemployed**	13.2%	14.6%	11.8%
**Symptom status (with symptoms)**	6.5%	4.6%	8.5%
**Development status**	4.49 (1.13)	4.27 (1.00)	4.71 (1.26)
**Individual behavior**	4.58 (2.24)	4.14 (2.08)	5.03 (2.41)

**Figure 3:**
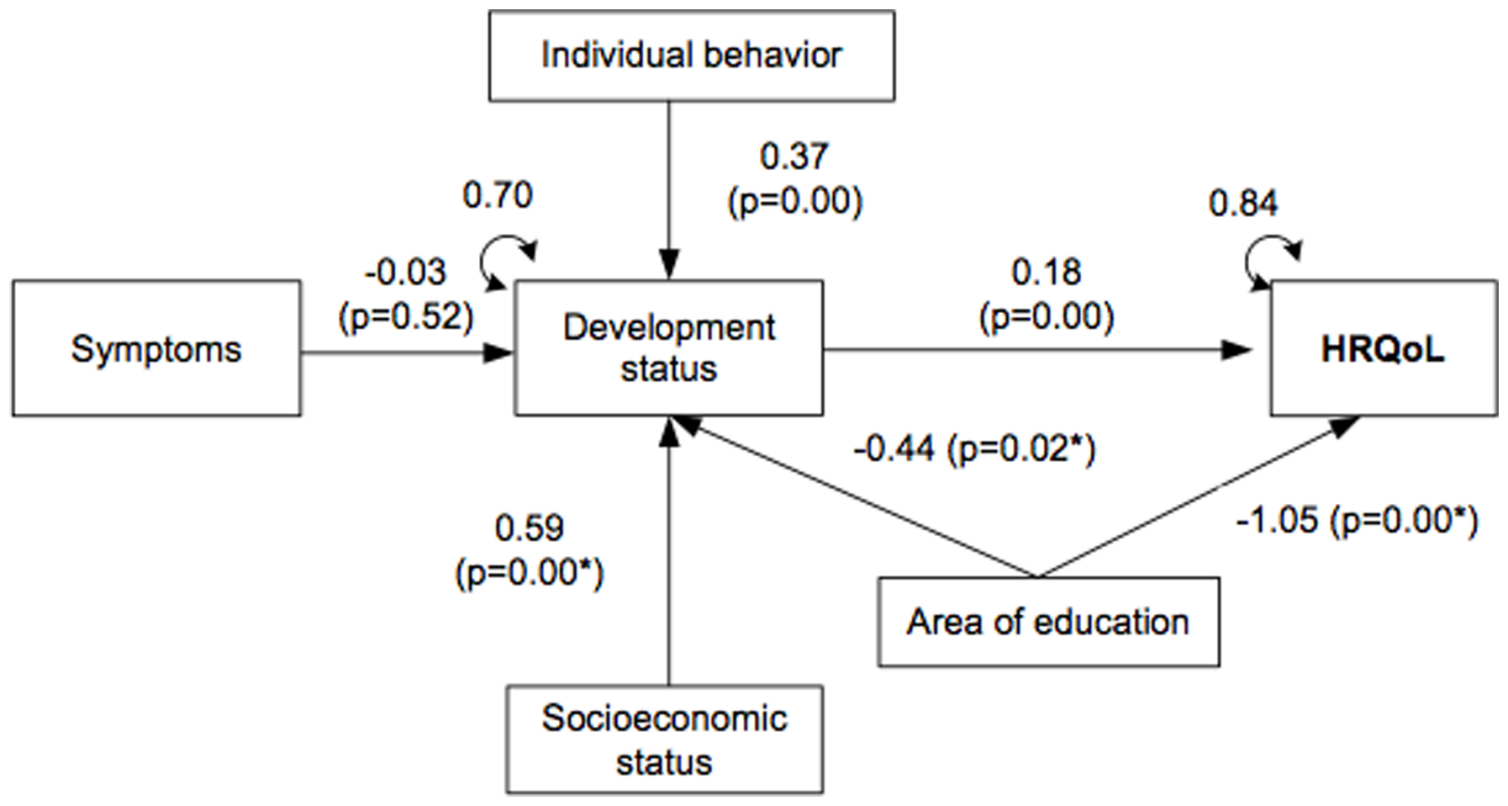
Measurement of variables and standardized estimates (β, ρ values and residual variances) of the test of the Wilson and Cleary theoretical framework using pediatric data. (Note: χ2 = 5.5; df = 6; p = 0.48; SRMR  = 0.01. Standardized coefficients are given. *P value and coefficients with the highest effect in categories of the dummy variables socioeconomic status and area of education.

Figure 3 shows the model factors that were hypothesized to affect HRQoL in children. Model fit was χ2 = 5.5; df = 6; p = 0.48; SRMR  = 0.01. The overall variance of HRQoL explained by the model was 15%. Socioeconomic status had a significant positive effect on children's functional status. Those children that were in households where the main sustainer of the family was categorized as professional or professional with advanced qualifications, which was the 28.3% of the sample, had respectively 0.59 and 0.43 higher standard deviations on children's functional status compared to the unemployed, indicating the protective effect of better socioeconomic status. Four areas of education had a significant decrement on HRQoL. The most important of these effects was of −1.05 (p = 0.00). Modification indices suggested the areas of education would have an effect on the developmental status. We included the path into the model after revision of its theoretical implications. This path was significant in one area of education, and indicated a significant negative effect of −0.44 (p = 0.20), and contributed to a better goodness of fit of the model. Symptoms did not have an effect on the developmental status. Regarding symptoms, despite the hypothesis, we considered the result normal, since only 6.5% of children suffered from a chronic disease or were hospitalized the week before the test. However, the variable remained in the model to maintain all the factors related to HRQoL in the hypothesized model. Individual behaviors showed a positive significant effect on the developmental status of 0.37 (p = 0.00). Then, an increment of one SD in individual behavior implies an increment of 0.37 SD in development status. And development status had a positive significant effect on HRQoL of 0.18 (p = 0.00). Then, an increment in one SD in development status implies an increment in 0.18 SD in HRQoL.

## Discussion

The goodness of fit of the model was good, indicating that our adaptation of the Wilson and Cleary theoretical framework can be used in children. Compared with previous studies that measured general HRQoL in children with adverse health conditions and diseases [18], [19], the variance explained of HRQoL was acceptable. However, the amount of variance explained suggests that factors that contribute to HRQoL in children are lacking in our model.

Our results confirm the relevance of developmental status and environmental factors as influences on HRQoL among kindergarten children from predominantly migrant backgrounds.

As we hypothesized, developmental status is an important variable directly associated with HRQoL [20]. Our test also supports the notion that individual behavior has an important effect on developmental status. It suggests that children's behaviors such as communication with peers, and social interactions in the kindergarten, among others, affect their development positively. Additional contextual and socioeconomic variables, viz., socioeconomic status and school-areas may also play an important role in child development. In the case of the contextual variables, these play an important role in different ways. First, higher socioeconomic position acts as a protective factor in our study. The relationship between the socioeconomic position of individuals and their health is well established – the socioeconomically better off perform better on most measures of health status, including HRQoL. The general pattern of better health among those who are socioeconomically better off is found across time periods, demographic groups, most measures of health and disease, as well as different measures of socioeconomic position [21], [22]. Second, the association between school-areas with HRQoL can be explained by several mechanisms. A contextual explanation posits that there are differences in the quality of the social or physical environment associated with different neighborhoods that influence the health of those exposed to them. More affluent neighborhoods are more likely to be associated with the provision of decent housing, safe playing areas, transport, green spaces and street lighting – all of which generate positive feelings about the community and leads to greater levels of social interaction and community participation; and consequently, better health related outcomes. Conversely when there is a lack of these characteristics, the impacts on health are correspondingly bad. Environmental design and layout can influence social interactions and the level of social cohesion [22], [23].

Sousa et al. performed one of the few studies that validated the Wilson and Cleary theoretical framework in a general adult population. To our knowledge, this is the unique report that uses structural equation modeling to do so [7], [24]. Despite the differences in population and methodology used in these studies, we believe that a comparison is illustrative in terms of the constructs that are missing with respect to the instruments used to measure HRQoL. The authors obtained good results in the model fit and explained 83% of the variance of overall HRQoL. The differences in results between this and our study can have several explanations. First, we performed our analyses including environmental factors and characteristics of the individual, while Sousa et al. did not include indicators of these concepts, but information about general health perceptions of adults was included. Second, the indicators used to test each construct differed strongly, as expected. For example, a specific scale to measure symptoms, as well as a disability index to assess functional status were included in the model of Sousa et al [7]. Interestingly, symptom status explained almost 49% of the variance in functional status in the Sousa et al. study. While in our study, the variance explained of functional status in our model was 31% and the contribution of symptoms was low. However, in children, the assessment had some differences. The sample was homogeneous. This means that in our sample, only the 6.5% of children reported having a chronic disease or having being recently hospitalized. Even though we consider this result is normal in pediatric populations, we probably lacked sufficient variation to observe the relationship between symptom status and functional status and a better indicator as it was assessed in the test with adult populations. In addition, our analysis was performed using comprehensive generic HRQoL measurement, and the test with the adult population investigated overall HRQoL and it comprised a disease specific population. It is also possible that fewer variables would explain more variance of overall HRQoL in a specific population than using generic HRQoL in general population. The questionnaire administered to quantify functional status had some variations. We included an indicator of functioning based on development, since the main difference of childhood is the rapid development in a physical, sensory-motor, mental, emotional and social dimension [9]. Sousa et al. assessed functioning based on the ICF suggesting that disability should be considering the level of functioning of a person [4]. However, our measure did not include an indicator of physical health or social functioning [9].

Apart from the differences we have enumerated between the Sousa et al. model test and ours, we consider there are essential differences between pediatric and adult questionnaires that can have had an influence in our results. During the development of the original theoretical framework, Wilson and Cleary stated that they were presenting a conceptual model, a taxonomy of patient outcomes, that categorizes measures of patient outcomes according to the underlying health concepts they represent and proposed specific causal relationships between the different health concepts. They then said that there is a conceptual distinction between identifying the dimensions of health that are necessary to comprehensively and validly describe health, versus specifying a series of critical concepts on a causal pathway [9], and that the latter was their main goal. Following these statements, in the literature there have been different attempts using the Wilson and Cleary theoretical framework: to test HRQoL itself, as opposed to identifying the contributing factors that are affecting HRQoL [25]. For example, in some cases, factors such as social well-being have been suggested to be contributing factors, predictors that would affect HRQoL, instead of being part of HRQoL itself. Whereas, on the contrary, social well-being has been part of HRQoL too. This means that, in the current debate, there could be some factors that exist both inside and outside the concept.

There are two examples that clearly reflect the mixed approaches in the measurement of HRQoL in children. Pediatric questionnaires that assess general HRQoL include “resilience” and “bullying” as part of HRQoL, and these are included as dimensions of general HRQoL [26], [27]. Yet resilience would also seem to be a determinant of HRQoL that is part of the characteristics of an individual, rather than a constituent dimension of HRQoL. Nevertheless, it has been targeted as a component of HRQoL. Bullying is another example of a factor that can clearly be a determinant of HRQoL which is part of the social context; however, it too has been used as a component of HRQoL [28]. This confusion between the concept and its determinants can lead to problems not only in assessing the model we test, but also in identifying outcomes and treatment [25]. A review of the literature reveals substantial heterogeneity in instruments for measuring HRQoL in children [29], which has been attributed to the paucity of theoretical grounding during the construction of these instruments. It has also been attributed to the lack of a common definition of HRQoL [29].

One of the most widely used measures to assess generic subjective health status in adults is the SF-36 which has been validated extensively. It is based on a multidimensional model of health and represents eight of the most important health domains, included in the Medical Outcomes Study and other commonly used health surveys [20], [30], [31]. The SF-36 captures dimensions with indicators that have a strong relationship with the Wilson and Cleary theoretical framework. Hence, multiple categories of operational definitions were chosen to investigate each health domain when the SF-36 was developed: behavioral functioning, perceived well-being, social and role disability, and personal evaluations and health in general.

To test the conceptual unity between the SF-36 and the KK, we mapped the dimensions of the KK onto the SF-36 dimensions considering the underlying concepts the SF-36 includes. Taking into account our previous premise that HRQoL components should have in common underlying concepts in adults and children measures, we suggest there should not be a lot of differences between the dimensions measured by the SF-36 and pediatric measures that assess general HRQoL.

This mapping however revealed some conceptual problems (Table 3). The indicators for HRQoL in both instruments are clearly different, because age determines the indicators that should be included to represent each concept, which are different in children compared to adults. However, not all the dimensions that are captured by the SF-36 are clearly reflected in the instrument we used. The mapping results indicate that general health is not reflected in the KK instrument, and that vitality and bodily pain are only partially reflected. However, these dimensions can be key points for children too. Children are normally healthy and report good general health and good levels of vitality, so it is especially important to detect those that have low levels of negative outcomes.

**Table 3:** Mapping the Kiddy-KINDL into SF-36 dimensions of HRQoL.

Underlying health domains of the SF-36	SF-36 dimensions	Kiddy-KINDL dimensions
**Behavioral functioning**	Physical functioning	Physical well-being
**Social and role disability**	Role physical	
**Social and role disability Perceived well-being**	Bodily pain	Partially related with Physical well-being items
**Personal evaluations and health in general**	General health	
**Perceived well-being**	Vitality*	Everyday functioning at the kindergarten, family, friends*
**Social and role disability**	Social functioning	Everyday functioning at the kindergarten, family, friends
**Social and role disability**	Role emotional*	Self-esteem*
**Perceived well-beingBehavioral functioning**	Mental health	Psychological well-being

Note: *The dimensions are not extremely connected.

Parts of the underlying concepts “personal evaluations and health in general”, and “social role and disability” are lacking in the questionnaire we used to test HRQoL. We have used the short version of the KINDL, the Kiddy-KINDL, which is more in accordance with the age of the kids of our sample; however the concepts are only partly reflected in the Kiddy-KINDL. Considering other measures of generic HRQoL for pediatric population, like the CHIP, the KIDSCREEN-52 or the GHQ, these underlying concepts are more represented in the dimension “self-perception” of the KIDSCREEN-52, and “satisfaction and diseases” of the CHIP questionnaire, and the CHQ has a wide relationship with the SF-36 underlying concepts [26], [27], [32]–[34]. Thus, caution is needed when generic HRQoL is under investigation in children, since several components have been related to it, and these vary considerably between questionnaires. Furthermore, the inclusion of “economic resources” as a dimension in a questionnaire that measures HRQoL like the KIDSCREEN-52 demonstrates again that some factors exist both inside and outside the concept. All this indicates substantial heterogeneity in the instruments for measuring HRQoL in children [29].

Some further limitations of our study deserve comment. First, our results are based on a kindergarten-based sample and results cannot be generalized. But we are confident that the results have high internal validity, and the relationships we have tested do not need participants be representative to drive our conclusions [35], [36]. Second, we only used cross-sectional baseline data, while longitudinal data are more appropriate to test a model using Structural Equation Modeling. Nevertheless, the variables included in the model followed the temporal sequence of the variables. Third, the sample size was small, and limited our statistical power, and the indicators we selected to test the model were those that we had available in our study and had some limitations, above all because we did not have indicators of each factor available in our study and because some indicators were poor, as in case of symptoms. However, we were able to include some indicators that were not included in previous validations of the theoretical framework and we have seen that despite this limitation, a number of relationships in the model are statistically significant and the variance explained of HRQoL is high.

The study also has several strengths. To our knowledge, this is the first study that investigates HRQoL in young kindergarten children via self-report; it also presents the first validation attempt of the Wilson and Cleary theoretical framework to use structural equation modeling in a pediatric population. And we used multiple informants considering the content of the measures we wanted to test.

In conclusion, the analysis implies that the relations depicted in the figures we present were supported by the data. This can be seen as an initial step in comprehensive testing of the Wilson and Cleary theoretical framework in a pediatric population and it suggests the framework can be largely used to test the contributors to HRQoL in children. However, our findings also underscore the need to study the influence of other factors to HRQoL, and to assess whether the variance explained increases. Our investigation suggests a conceptual gap between adult and children measures, and a great variation in the assessments of children's HRQoL. There is a common view of the multidimensionality of HRQoL, and that the construct of health is viewed differently by children in comparison to adults [1]. But it appears that instruments of generic HRQoL in children are based on distinct definitions. We suggest that pediatric measures need to add indicators, determinants or predictors that are more related to a common definition of HRQoL. Hence, given how difficult is to conceptualize HRQoL in children, we recommend that pediatric HRQoL researchers invest more time developing a common perspective of which are the basic dimensions that should be tested in pediatric populations.
